# Circulating Genetically Abnormal Cells Add Non-Invasive Diagnosis Value to Discriminate Lung Cancer in Patients With Pulmonary Nodules ≤10 mm

**DOI:** 10.3389/fonc.2021.638223

**Published:** 2021-03-11

**Authors:** Maosong Ye, Xiaoxuan Zheng, Xin Ye, Juncheng Zhang, Chuoji Huang, Zilong Liu, Meng Huang, Xianjun Fan, Yanci Chen, Botao Xiao, Jiayuan Sun, Chunxue Bai

**Affiliations:** ^1^Department of Pulmonary Medicine, Zhongshan Hospital, Fudan University, Shanghai, China; ^2^Department of Respiratory Endoscopy, Shanghai Chest Hospital, Shanghai Jiao Tong University, Shanghai, China; ^3^Department of Respiratory and Critical Care Medicine, Shanghai Chest Hospital, Shanghai Jiao Tong University, Shanghai, China; ^4^Joint Research Center of Liquid Biopsy in Guangdong, Hong Kong, and Macao, Zhuhai, China; ^5^Zhuhai Sanmed Biotech Ltd., Zhuhai, China; ^6^School of Biology and Biological Engineering, South China University of Technology, Guangzhou, China

**Keywords:** lung cancer, pulmonary nodule, early detection, early diagnosis, circulating genetically abnormal cell (CAC)

## Abstract

**Background:**

Lung cancer screening using low-dose computed tomography (LDCT) often leads to unnecessary biopsy because of the low specificity among patients with pulmonary nodules ≤10 mm. Circulating genetically abnormal cells (CACs) can be used to discriminate lung cancer from benign lung disease. To examine the diagnostic value of CACs in detecting lung cancer for patients with malignant pulmonary nodules ≤10 mm.

**Methods:**

In this prospective study, patients with pulmonary nodules ≤10 mm who were detected at four hospitals in China from January 2019 to January 2020 were included. CACs were detected using fluorescence *in-situ* hybridization. All patients were confirmed as lung cancer or benign disease by further histopathological examination. Multivariable logistic regression models were established to detect the presence of lung cancer using CACs and other associated characteristics. Receiver operating characteristic analysis was used to evaluate the performance of CACs for lung cancer diagnosis.

**Results:**

Overall, 125 patients were included and analyzed. When the cutoff value of CACs was >2, the sensitivity and specificity for lung cancer were 70.5 and 86.4%. Male (OR = 0.330, P = 0.005), maximum solid nodule (OR = 2.362, P = 0.089), maximum nodule located in upper lobe (OR = 3.867, P = 0.001), and CACs >2 (OR = 18.525, P < 0.001) met the P < 0.10 criterion for inclusion in the multivariable models. The multivariable logistic regression model that included the dichotomized CACs (>2 *vs.* ≤2) and other clinical factors (AUC = 0.907, 95% CI = 0.842–0.951) was superior to the models that only considered dichotomized CACs or other clinical factors and similar to the model with numerical CACs and other clinical factors (AUC = 0.913, 95% CI = 0.850–0.956).

**Conclusion:**

CACs presented a significant diagnostic value in detecting lung cancer for patients with pulmonary nodules ≤10 mm.

## Introduction

Lung cancer is the leading cause of mortality globally, and it is important to detect lung cancer in early-stage to improve patients’ survival and life quality (1, 2). The main issue with low-dose computed tomography (LDCT) in lung cancer screening is the high false-positive rate, which is often difficult for physicians to conclude whether their patients can be benefited from additional invasive examinations, including biopsy or surgery (3). Particularly, among those high-risk individuals with pulmonary nodules ≤10 mm, about 1/3 is benign (3, 4). Therefore, individuals who have benign pulmonary nodules or small nodules (≤10 mm) are at risk of being misdiagnosed by LDCT. Meanwhile, the sensitivity and specificity of current non-invasive serum tumor biomarker, such as CEA, NSE, CYFR21-1, could not meet the needs of lung cancer diagnosis at an early stage (5–7). Currently, specific non-invasive tests with the ability to maximize the accuracy and reduce potential associated harms are warranted in urge (3, 4).

In 2010, MD Anderson Center developed a fluorescence *in-situ* hybridization (FISH)-based detection method for the detection of circulating genetically abnormal cells (CACs) (8). The presence of this type of cells is due to the inability of cancer cells to preserve the structure of chromosomes during replication (9), leading to the rearrangements and aneusomy (10–12). Chromosomal instability is found in 9% of the premalignant lesions and 59% of the malignant lesions in non-small cell lung cancer (NSCLC) (13). Additionally, Katz and her colleagues found frequent abnormalities of these genes in the sputum and resected tissue specimens obtained from patients with NSCLC. The presence of CACs in the blood is an early event in the pathogenesis of lung cancer (8, 14). A recent study from MD Anderson showed that the CACs yielded 94% accuracy, 89% sensitivity, and 100% specificity compared with biopsy for diagnosing lung cancer (14). Nevertheless, the value of CACs was not specifically tested for individuals with pulmonary ≤10 mm.

In light of these researches, it is possible that CACs can be used in detecting lung cancer. However, since CACs have not been specifically evaluated for individuals with pulmonary nodules ≤10 mm, the diagnostic effect of CACs was still worth testing in those patients to avoid the need to conduct invasive approaches. The aim of this study was to examine the diagnostic value of CACs in detecting lung cancer for patients with malignant pulmonary nodules ≤10 mm.

## Materials and Methods

### Study Design and Population

The present multicenter, prospective, diagnostic study was within the Multicenter Chinese Pulmonary Nodule Detection (MCPND) project, which aimed at differentiating benign from malignant pulmonary nodules. All participants with pulmonary nodules ≤10mm were enrolled at Shanghai Chest Hospital, Zhongshan Hospital affiliated to Fudan University, Suining Central Hospital, Sun Yat-sen Memorial Hospital of Sun Yat-sen University, and Tianjin Medical University Cancer Institute and Hospital in China from January 2019 to January 2020.

The inclusion criteria for the study were: 1) ≥18 years of age; 2) with single or multiple pulmonary nodules ≤10 mm detected by thin-slice chest CT within the last 6 months; 3) planned for non-surgical biopsy or surgical resection of pulmonary nodules with a histopathological examination; and 4) agreed to participate in the study and signed the informed consent forms. The exclusion criteria were: 1) female patients who were breastfeeding, pregnant, or preparing to become pregnant; 2) with severe heart, lung, liver, and kidney dysfunction, or mental disorders; 3) previously received lung cancer-related clinical therapeutic interventions such as surgery, radiotherapy, chemotherapy, targeted therapy, or immunotherapy; or 4) history of malignancy within the last 5 years. This study was approved by all the Ethics Committee of the participating hospitals. All patients signed the informed consent prior to any study procedure.

### CAC Detection

Peripheral blood (10 ml) was collected preoperatively for CAC detection once were patient enrolled. Chromosomes 3 and 10 [probes for 3p22.1/3q29 (196F4), and 10q22.3/CEP10] abnormalities of the peripheral blood mononuclear cells (PBMCs) were qualitatively detected using the Single Nucleus Cell Chromosome Abnormality Detection Kit (Zhuhai Sanmed Biotechnology Ltd., Zhuhai, China) and FISH. The PBMCs were counted and adjusted to 40,000 cells/100 µl. They were deposited as cytospins, spray-fixed with alcohol, and stored at −20°C. Fluorescent probe complexes were created by FISH using a set of four probes: 3q29 (196F4), 3p22.1, CEP10, and 10q22.3 (8, 14). Finally, the nuclei were stained with 4′,6-diamidino-2-phenylindole (DAPI).

The processed samples were analyzed using the Duet System (Allegro plus, BioView, Ltd., Rehovot, Israel), a pathological section scanner, to identify and detect the numbers of abnormal chromosomes 3 and 10. The signal distributione of the chromosomally abnormal cells shows increased or loss of chromosomal sites. Cells with increased polysomy (i.e., two probes of chromosome 3 with three or more signals, or two probes of chromosome 10 with three or more signals) were identified as CACs.

### Serum Tumor Markers in Lung Cancer

Tumor biomarkers, carcinoembryonic antigen (CEA), squamous cell carcinoma antigen (SCC), neuron-specific enolase (NSE), pro-gastrin-releasing protein (ProGRP), and cytokeratin fragment (CYFRA) 21-1 were measured once patient were enrolled. A 3-ml anticoagulated peripheral blood sample was collected from all participants. On the day of sample collection, electrochemiluminescence immunoassay (ECLIA) was applied to detect the serum levels of the tumor biomarkers. We also considered a combined situation as “any biomarker” positive whenever any of the above biomarkers was tested positive.

### Gold Standard

The histopathological classification in this study was based on the 2015 WHO Histological Classification of Lung Cancer (15). Biopsy or postoperative pathological examination was performed within 5 working days after the CACs test. All pulmonary nodules were classified into malignant and benign nodules.

### Data Collection

Baseline data (age, sex, and smoking history) were collected and analyzed. The thin-slice chest CT examination was performed using a multidetector CT machine. The size of the maximum nodule, the type of the maximum nodule (solid and non-solid), and the location of the maximum nodule (left lung, right lung, upper lobe, and non-upper lobe) were also recorded.

### Statistical Analysis

Descriptive data were expressed in number and percentage for categorical variables. The chi-square test was used to compare categorical variables between the groups. For continuous variables that fit a normal distribution, means ± standard deviations (SD) were presented; otherwise, medians and interquartile ranges (25th–75th percentile) were reported. Independent samples t-test and Mann-Whitney U test for continuous variables were applied, respectively.

The sensitivity, specificity, and Youden index of each biomarker were calculated. The receiver operating characteristic (ROC) curve and the area under the curve (AUC) were used to assess the predictive effect. The comparison of the AUCs was based on the method of DeLong et al. (16). Univariable and multivariable logistic regression analyses were performed to evaluate the significant factors associated with malignant pulmonary nodules. Variables with P < 0.10 in the univariable logistic regression were included in the multivariable logistic regression. Then final prediction models were constructed by the backward method. The variance inflation factor (VIF) was used to identify collinearity among the covariables. We estimated the goodness of fit for the final model using a Hosmer and Lemeshow test. Two-sided P-values <0.05 were considered statistically significant. Statistical analyses were conducted using MedCalc (MedCalc Software Ltd, Ostend, Belgium) and SPSS 22.0 (IBM, Armon, NY, USA).

## Results

### Characteristics of the Participants

Of the 420 individuals who were enrolled in the MCPND trials, we excluded patients whose pulmonary nodule was bigger than 10 mm (**Supplemental Figure 1**). A total of 125 patients were included in our analyses, of which 47 (37.6%) were male, and 78 (62.4%) were female. Eighty-one (64.8%) participants were confirmed with lung cancer, and the other 44 (35.2%) were benign. All of them have the tests result of CACs and tumor markers.

Compared with patients who had benign nodules, individuals with lung cancer had a lower proportion of males (28.4 *vs.* 54.5%, P = 0.004), a lower frequency of smoking history (14.3 *vs.* 38.6%, P = 0.008), their maximum nodules are more likely to appear in the upper lobe (66.7 *vs.* 34.1%, P < 0.001), and a higher number of CACs (Median: 4 *vs.* 1, P < 0.001). There were no differences in age, nodule size, type of nodule, late rality, and tumor markers (all P > 0.05, **Table 1**).

**Table 1:** Characteristics of the participants.

Variables	Benign (n = 44)	Malignant (n = 81)	P
Age, year	53.7 ± 10.0	50.9 ± 12.4	0.076
Sex (male), n (%)	24 (54.5%)	23 (28.4%)	0.004
Smoking history, n (%)	17 (38.6%)	14 (14.3%)	0.008
Size of the maximum nodule, mm	8 (7,9)	8 (7,10)	0.294
Type of the maximum nodule, n (%)			0.083
Solid	6 (13.6%)	22 (27.2%)	
Non-solid	38 (86.4%)	59 (72.8%)	
Location of the maximum nodule, n (%)			
Right/Left			0.703
Right lung	26 (59.1%)	45 (55.6%)	
Left lung	18 (40.9%)	36 (44.4%)	
Upper/Non-upper			<0.001
Upper lobe	15 (34.1%)	54 (66.7%)	
Non-upper lobe	29 (65.9%)	27 (33.3%)	
Preoperative CAC, Median (interquartile)	1 (2)	4 (4)	<0.001
CEA positive, n (%)	5 (11.4%)	6 (7.4%)	0.678
SCC positive, n (%)	0 (0%)	6 (7.4%)	–
NSE positive, n (%)	6 (13.6%)	8 (9.9%)	0.734
Pro-GRP positive, n (%)	3 (6.8%)	3 (3.7%)	0.734
CYFRA21-1 positive, n (%)	5 (11.4%)	11 (13.6%)	0.723

CAC, circulating genetically abnormal cells; CEA, carcinoembryonic antigen; SCC, squamous cell carcinoma antigen; NSE, neuron-specific enolase; CYFRA21-1, cytokeratin fragment 21-1.

### CACs and Tumor Markers for the Diagnosis of Lung Cancer

**Figure 1:**
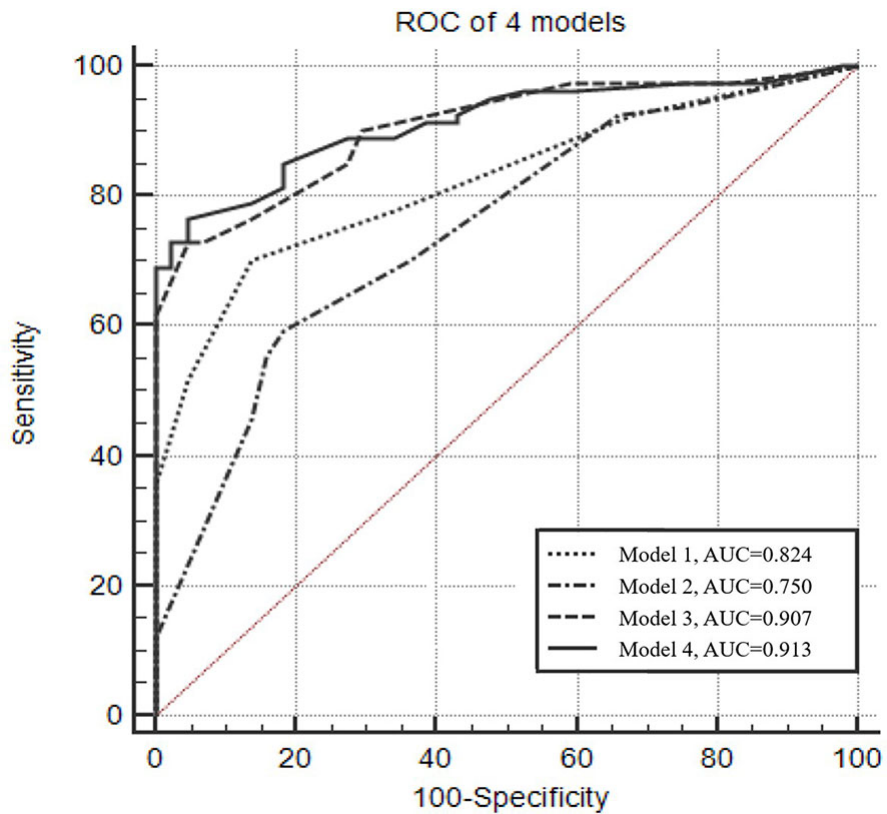
Receiver operating characteristic curve of the four predictive models for diagnosing lung cancer. Model 1: Numerical CACs. The optimal Youden index was observed when CACs equals 2, with 70.4% sensitivity and 86.4% specificity. Model 2: Male, type of the maximum nodule (solid), location of the maximum nodule (upper lobe). Model 3: Dichotomized CACs (cutoff value >2), male, type of the maximum nodule (solid), location of the maximum nodule (upper lobe). Model 4: Numerical CACs, male, type of the maximum nodule (solid), location of the maximum nodule (upper lobe).

The optimal Youden index for CACs was observed at a cutoff value of >2, resulting in 70.4% sensitivity and 86.4% specificity (**Figure 1**). The diagnostic efficacy of the CACs (AUC = 0.824) was significantly higher than that of CEA, SCC, NSE, Pro-GRP, CYFRA 21-1, and any biomarker (AUC = 0.520, 0.537, 0.519, 0.516, 0.511, and 0.512, respectively) (**Table 2**).

**Table 2:** ROC curve analysis of CACs and biomarkers.

Variables	AUC	95% CI
CACs	0.824	0.746–0.886
CEA	0.520	0.429–0.610
SCC	0.537	0.446–0.627
NSE	0.519	0.428–0.609
Pro-GRP	0.516	0.425–0.606
CYFRA21-1	0.511	0.420–0.602
Any biomarker	0.512	0.421–0.602

AUC, area under the curve; CAC, circulating genetically abnormal cells; CEA, carcinoembryonic antigen; SCC, squamous cell carcinoma antigen; NSE, neuron-specific enolase; pro-GRP, pro-gastrin-releasing peptide; CYFRA21-1, cytokeratin fragment 21-1.

### Regression Models

**Figure 2:**
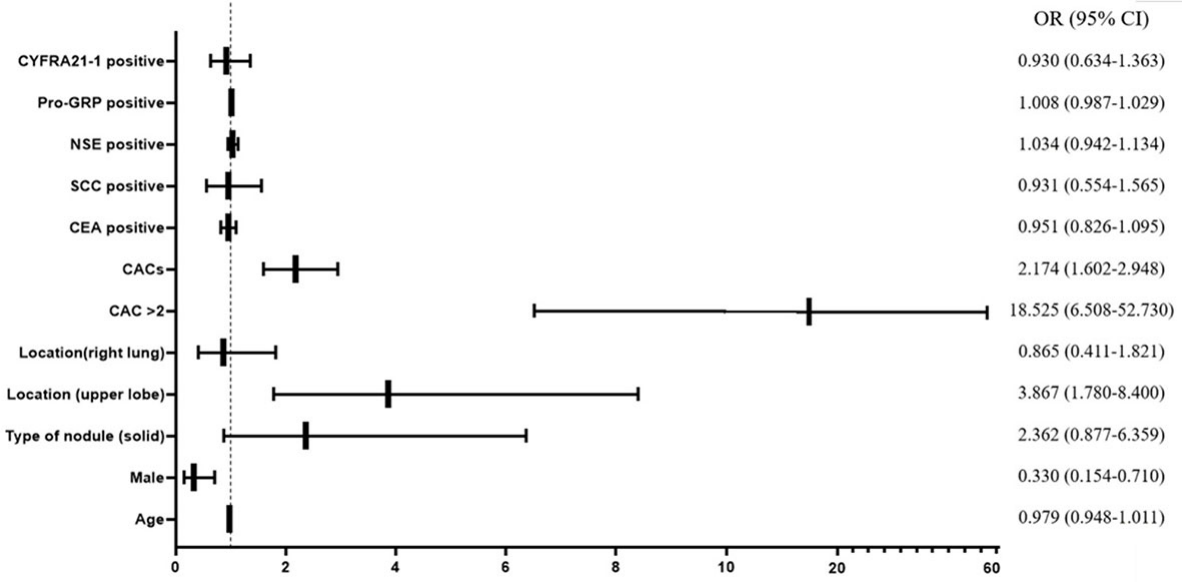
Forest plot of univariable logistic regression analysis. CAC, circulating genetically abnormal cells; CEA, carcinoembryonic antigen; SCC, squamous cell carcinoma antigen; NSE, neuron-specific enolase; pro-GRP, pro-gastrin-releasing peptide; CYFRA21-1, cytokeratin fragment 21-1.

To further examine if the CACs and other clinical characteristics could significantly affect the diagnosis of lung cancer in discriminating malignant nodule from a benign nodule, we performed the univariable logistic regression to assess the potential risk factors. Consequently, male gender (OR = 0.330, 95% CI = 0.154–0.710, p = 0.005), solid maximum nodule (OR = 2.362, 95% CI = 0.877–6.359, P = 0.089), maximum nodule located in upper lobe (OR = 3.867, 95% CI = 1.780–8.400, P = 0.001), CACs >2 (OR = 18.525, 95% CI = 6.508–52.73, P < 0.001), and numerical CAC (OR = 2.174, 95% CI = 1.602–2.948, P < 0.001) were associated with malignant nodule at P < 0.10 (which was the criterion for entry in the multivariable models) (**Figure 2**, **Supplemental Table 1**). Subsequently, we performed a multivariable logistic regression analysis to further determine the influence of CACs as well as significant clinical characteristics.

**Figure 3:**
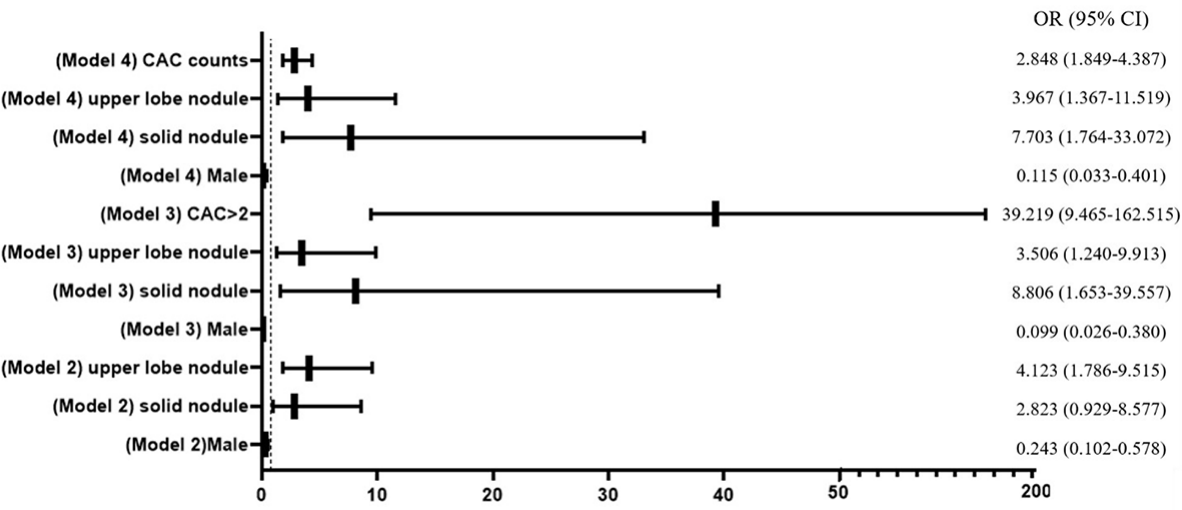
Forest plot of multivariable logistic regression analysis. CAC, circulating genetically abnormal cells; CEA, carcinoembryonic antigen; SCC, squamous cell carcinoma antigen; NSE, neuron-specific enolase; pro-GRP, pro-gastrin-releasing peptide; CYFRA21-1, cytokeratin fragment 21-1.

Three multivariable logistic regression models, which considered significant clinic factors and dichotomized CACs with the cutoff value of >2 or numerical CACs, were established to predict the probability of malignant nodule (**Figure 3**). The ability of the three models in discriminating malignant nodule from benign nodule was evaluated by C-index (AUC).

**Figure 1** presents the ROC analyses of different models. The AUC of the model that only considered numerical CACs was 0.824, and the model that only considered clinical factors (gender, type of the maximum nodule, location of the maximum nodule) was 0.750. However, when the clinical factors and the dichotomized CACs were considered together, the AUC was 0.907. Results from ROC also indicated the AUC could reach 0.913 when the dichotomized CACs were replaced by the numerical CACs in the combined model (**Figure 1**, **Supplemental Table 2**).

## Discussion

In this study, we examined the diagnostic value of CACs in detecting lung cancer for patients with pulmonary nodules ≤10 mm. The results strongly suggested that the combination of either the dichotomized or numerical CACs with other clinical risk factors could significantly improve the prediction of lung cancer in patients with pulmonary nodules ≤10 mm. CACs could be used as an additive indicator for the indeterminately radiographic approach to detect patients with pulmonary nodules ≤10 mm in early lung cancer screening.

To date, the detection of lung cancer for patients with pulmonary nodules ≤10 mm remains a diagnostic challenge (17–20). When the size of the pulmonary nodule is less than 10 mm, due to the low specificity of CT, clinicians were facing the dilemma of whether performing additional imaging and even an invasive procedure or not (17–19). During the early stage of lung cancer, cytogenetically abnormal cells can be detected in the bloodstream and sputum (8, 14). The detected abnormal cell distinctively displayed the chromosomal abnormalities (10–12) and genetic abnormalities in tumor suppressor genes and proto-oncogene (21, 22), which are common in patients with lung cancer. Therefore, in early lung cancer screen, specifically detecting CACs could possibly yield a higher auxiliary diagnosis value compared to circulating tumor cells (CTCs). Since CTCs are characterized by surface markers, the sensitivity will be greatly reduced if the markers of CTCs are undetectable (23).

The present study suggested CACs >2 could yield a 70% of sensitivity and 86% of specificity with an AUC of 0.824, differentiating the pulmonary nodules between malignant and benign, which indicated better effectiveness in diagnosing lung cancer compared to traditional biomarkers. This result is similar to a recent study conducted by Katz et al. investigating the diagnostic value of CACs for lung cancer in patients, irrespective of the size of the pulmonary nodules. The results of their study showed 89% of sensitivity and 100% of specificity for diagnosing lung cancer when using ≥3 CACs (14). Based on the demographic information in their research, patients with large nodules had a higher probability of being malignant, while patients with pulmonary nodules <10 mm had a higher probability of being benign, which probably explained the relatively lower sensitivity and specificity observed only use CACs in our study (14).

When using either numerical or dichotomized CACs in combination with other significant variables, AUCs >0.9 were achieved, which represented an excellent auxiliary diagnostic value of CACs. The model that combined numerical CACs and clinical factors (gender, type of the maximum nodule, location of the maximum nodule) exhibited the highest AUC in our study (AUC = 0.913). The Mayo model is a well-recognized model that was commonly used in the screening of lung cancer for patients who had an uncertain pulmonary nodule under 3 cm found by radiographic approach without tissue diagnosis (24). This model included smoking history, age, nodule size, and the time from quit smoking, has achieved an AUC of 0.79 (25). Both of our model and the Mayo model include clinical factors, but our results indicated that taking into account CACs could possibly improve the diagnostic effect in detecting malignant pulmonary nodules in the case when nodules were small (≤10 mm). In terms of the patients’ aspects, as the time from misdiagnosis lasting, the best therapeutic time window might be ignored. In contrast, long-term follow-up and multiple CT examinations can induce anxiety. What’s more, under the application of a non-invasive method in the diagnosis of lung cancer, the harm from invasive procedures can be avoided. Therefore, using CACs as a predictor can possibly improve the quality of the management and prevent patients from harm and psychological burden by using a simple blood sample instead of biopsy.

Our study suffered from several limitations. Firstly, only a few patients in the MCPND project with nodules ≤10 mm were willing to undergo biopsy and surgical resection, resulting in a relatively small sample size and the lower limit of 7 mm in this study, which might influence the validation of our conclusion in patients with pulmonary nodule <7mm. Moreover, non-surgical biopsy has a false positive rate of 30–40%, according to the literature (26, 27). For patients whose non-surgical biopsy show negative results while the CACs test and the model in our study show positive results, we suggest a close follow-up, such as shortening the CT follow-up period or a second biopsy. Secondly, due to the positive involvement of CT screening in early-stage females, a higher proportion of females was enrolled, which also resulted in a lower proportion of patients without smoking history in the malignant group. These limitations largely account for the existence of selection and referral biases. Moreover, considering the ethical reasons, only patients who had the demands of biopsy and surgery were included in the project, which resulted in a high proportion of malignant nodules. Nevertheless, this limitation is inherent to any study that designed with histopathological confirmation of the lesion. Therefore, future studies can further include patients whose malignant pulmonary nodules were diagnosed through the follow-up inspection.

In conclusion, with the presence of either numerical or dichotomized CACs, lung cancer can be effectively diagnosed in patients whose pulmonary nodules ≤10 mm. The model that considered both CACs and other clinic factors (gender, type of the maximum nodule, and location of the maximum nodule) could improve the effectiveness of lung cancer diagnosis and prevent patients from undergoing an unnecessary invasive procedure.

## Data Availability Statement

The raw data supporting the conclusions of this article will be made available by the authors, without undue reservation.

## Ethics Statement

The studies involving human participants were reviewed and approved by the ethics committee of Zhongshan Hospital Affiliated to Fudan University (b2019-185r). The patients/participants provided their written informed consent to participate in this study.

## Author Contributions

MY, XZ, XY and JZ have contributed equally in carrying out the studies. JS and CB contributed to the conception, study design, and administrative support. MY, XZ, and ZL contributed to the provision of study materials or patients. MY, XZ, CH and BX contributed to the data collection and assembly. MH, XF, and YC contributed to the data analysis and interpretation. MY, XZ, XY, and JZ contributed to drafting and revising the manuscript. All authors contributed to the article and approved the submitted version.

## Funding

This study was funded by the program for Guangdong Introducing Innovative and Entrepreneurial Teams (2019ZT08Y297).

## Conflict of Interest

XY, JZ, CH, MH, XF and YC was employed by Zhuhai Sanmed Biotech Ltd.

The remaining authors declare that the research was conducted in the absence of any commercial or financial relationships that could be construed as a potential conflict of interest.
