# Supplementary material for: Circulating Genetically Abnormal Cells Add Non-Invasive Diagnosis Value to Discriminate Lung Cancer in Patients With Pulmonary Nodules ≤10 mm
Source: Front Oncol. 2021 Mar 11;11:638223. doi: 10.3389/fonc.2021.638223 (PMC7991838; doi:10.3389/fonc.2021.638223)
Supplement: Supplementary file 1 [file DataSheet_1.docx]

***Supplementary Material***

1. **Supplementary Figures and Tables**

**Supplementary Tables**

**Supplementary Table 1.** Univariable logistic regression analysis

| Variables | OR | 95% CI | P |
| --- | --- | --- | --- |
| Age | 0.979 | 0.948-1.011 | 0.197 |
| Male | 0.330 | 0.154-0.710 | 0.005 |
| Type of the maximum nodule (solid) | 2.362 | 0.877-6.359 | 0.089 |
| Location of the maximum nodule (upper lobe) | 3.867 | 1.780-8.400 | 0.001 |
| Location of the maximum nodule (right lung) | 0.865 | 0.411-1.821 | 0.703 |
| CAC >2 | 18.525 | 6.508-52.730 | <0.001 |
| CACs | 2.174 | 1.602-2.948 | <0.001 |
| CEA positive | 0.951 | 0.826-1.095 | 0.485 |
| SCC positive | 0.931 | 0.554-1.565 | 0.788 |
| NSE positive | 1.034 | 0.942-1.134 | 0.487 |
| Pro-GRP positive | 1.008 | 0.987-1.029 | 0.478 |
| CYFRA21-1 positive | 0.930 | 0.634-1.363 | 0.709 |

CAC: circulating genetically abnormal cells; CEA: carcinoembryonic antigen; SCC: squamous cell carcinoma antigen; NSE: neuron-specific enolase; pro-GRP: pro-gastrin-releasing peptide; CYFRA21-1: cytokeratin fragment 21-1.

**Supplementary Table 2.** Multivariable logistic regression analysis for different models

|  | OR | 95% CI | P |
| --- | --- | --- | --- |
| Model 2 |  |  |  |
| Male | 0.243 | 0.102-0.578 | 0.001 |
| Type of the maximum nodule (solid) | 2.823 | 0.929-8.577 | 0.067 |
| Location of the maximum nodule (upper lobe) | 4.123 | 1.786-9.515 | 0.001 |
| Model 3 |  |  |  |
| Male | 0.099 | 0.026-0.38 | 0.001 |
| Type of the maximum nodule (solid) | 8.086 | 1.653-39.557 | 0.010 |
| Location of the maximum nodule (upper lobe) | 3.506 | 1.240-9.913 | 0.018 |
| CAC >2 | 39.219 | 9.465-162.515 | <0.001 |
| Model 4 |  |  |  |
| Male | 0.115 | 0.033-0.401 | 0.001 |
| Type of the maximum nodule (solid) | 7.703 | 1.794-33.072 | 0.006 |
| Location of the maximum nodule (upper lobe) | 3.967 | 1.367-11.519 | 0.011 |
| CACs | 2.848 | 1.849-4.387 | <0.001 |

CAC: circulating genetically abnormal cells.

**Supplementary Table 3.** Predictive value of different models

|  | AUC | 95% CI |
| --- | --- | --- |
| Model 1. Numerical CACs | 0.824 | 0.746-0.886 |
| Model 2. Clinical factors | 0.750 | 0.664-0.823 |
| Model 3. Dichotomized CACs and clinical factors | 0.907 | 0.842-0.951 |
| Model 4. Numerical CACs and clinical factors | 0.913 | 0.850-0.956 |

Clinical factors including gender, type of nodule, and location of nodule. AUC: area under the curve; CAC: circulating genetically abnormal cells.

**Supplementary Figure**


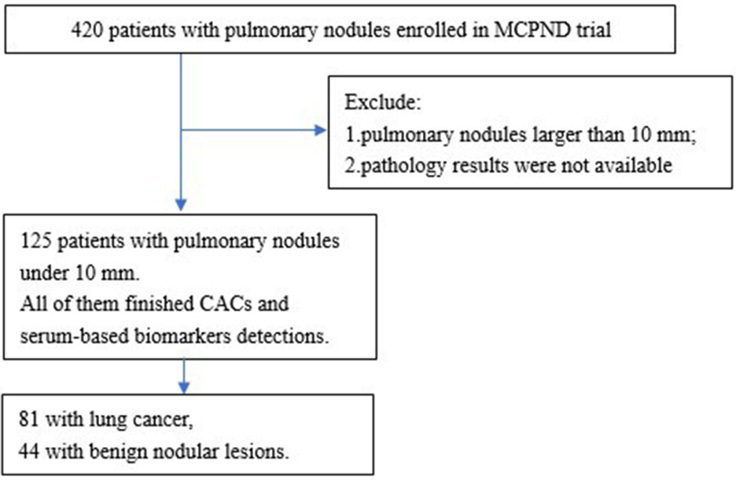


**Supplementary Figure 1.** Flowchart for patients’ enrollment.
